# Gene promoters show chromosome-specificity and reveal chromosome territories in humans

**DOI:** 10.1186/1471-2164-14-278

**Published:** 2013-04-24

**Authors:** Paul Gagniuc, Constantin Ionescu-Tirgoviste

**Affiliations:** 1 Institute of Genetics, University of Bucharest, Bucharest, 060101, Romania; 2 National Institute of Diabetes, Nutrition and Metabolic Diseases ”N.C. Paulescu”, Bucharest, Romania

## Abstract

**Background:**

Gene promoters have guided evolution processes for millions of years. It seems that they were the main engine responsible for the integration of different mutations favorable for the environmental conditions. In cooperation with different transcription factors and other biochemical components, these regulatory regions dictate the synthesis frequency of RNA molecules. Predominantly in the last decade, it has become clear that nuclear organization impacts upon gene regulation. To fully understand the connections between *Homo sapiens* chromosomes and their gene promoters, we analyzed 1200 promoter sequences using our Kappa Index of Coincidence method.

**Results:**

In order to measure the structural similarity of gene promoters, we used two-dimensional image-based patterns obtained through Kappa Index of Coincidence (Kappa IC) and (C+G)% values. The center of weight of each promoter pattern indicated a structure similarity between promoters of each chromosome. Furthermore, the proximity of chromosomes seems to be in accordance to the structural similarity of their gene promoters. The arrangement of chromosomes according to Kappa IC values of promoters, shows a striking symmetry between the chromosome length and the structure of promoters located on them. High Kappa IC and (C+G)% values of gene promoters were also directly associated with the most frequent genetic diseases. Taking into consideration these observations, a general hypothesis for the evolutionary dynamics of the genome has been proposed. In this hypothesis, heterochromatin and euchromatin domains exchange DNA sequences according to a difference in the rate of Slipped Strand Mispairing and point mutations.

**Conclusions:**

In this paper we showed that gene promoters appear to be specific to each chromosome. Furthermore, the proximity between chromosomes seems to be in accordance to the structural similarity of their gene promoters. Our findings are based on comprehensive data from Transcriptional Regulatory Element Database and a new computer model whose core is using Kappa index of coincidence.

## Background

Inside the body, somatic cells exercise their overall functions in G_0_ phase (the period between cell divisions) [1-3]. During this phase, individual chromosomes are impossible to distinguish by light or electron microscopy. For instance, when cells are terminally differentiated, some of them enter in a permanent (quiescent state) G_0_ phase, such as myocyte cells, the majority of neuronal cell types or pancreatic beta cells. Other types of cells exhibit a temporary G_0_ phase, such as glial cells or hepatocyte cells, which divide under controlled conditions. However, less is known of the precise location of chromosomes and their relationship with the internal nuclear membrane and nuclear pores through which the traffic of molecules is made. Inside the nucleus of specialized cells, spatial arrangements of chromosomes in G_0_ phase play an important role in the regulation of gene expression patterns [4,5]. The nucleus lacks of membrane compartmentalization [6,7]. In telophase, mitotic chromosomes unfold into chromatin state [8,9]. Immediately after nuclear membrane is formed, heterochromatin is allocated to the nuclear periphery whereas euchromatin is generally contained towards the nuclear interior. In G_0_ phase, chromatin shows different states of condensation, such as constitutive heterochromatin, facultative heterochromatin and euchromatin [10,11]. Constitutive heterochromatin consists of permanently condensed DNA, usually containing multiple short repeats and low gene density. Facultative heterochromatin represents a temporary DNA condensation state, located in heterochromatin landscape surface [12,13]. The active part of the nucleus (gene rich areas), where the transcription of DNA to mRNA is made, is represented by euchromatin domain. In order to initiate the transcription process, the relaxed structure of euchromatin allows regulatory proteins and RNA polymerase complexes to bind to DNA for transcription initiation and elongation of mRNA [14]. Euchromatin domains which are never stored as facultative heterochromatin are usually under active transcription and contain housekeeping genes, otherwise crucial for basic cell functions [15]. Genes embedded inside facultative heterochromatin can transit to and from euchromatin, depending on different functions that the cell needs to perform, in certain time intervals or under the action of certain external stimuli. It is recognized that many active genes that are brought into or near heterchromatin landscapes become repressed and their transcriptional reactivation is made by reallocation to the nuclear interior [16-18]. Nevertheless, other studies show that some genes are transcriptionally active close to nuclear periphery [19-21]. Electron microscopy images show a lack of heterchromatin around nuclear pores [22]. Although active inside euchromatin, some inducible genes from the nuclear interior are relocated near nuclear pores for a fast response under the action of certain stimuli [23-27]. However, facultative heterochromatin represents one of many methods through which cells, start or stop the expression of certain genes. Heterochromatin is also critical in morphogenesis and differentiation. In embryogenesis, chromatin establishes different structural landscapes depending on cell specialization. For instance, Hox gene clusters [28,29] are responsible for the spatial structure of the body. In humans, these genes are located on chromosome 7 (HOXA gene clusters), 17 (HOXB gene clusters), 12 (HOXC gene clusters) and 2 (HOXD gene clusters). In embryogenesis, Hox genes are brought to the surface into euchromatin domain in order to be expressed in a sequential manner [30,31]. Polycomb-group proteins and other biochemical mechanisms reshape chromatin depending on the cell type, allowing a favorable positioning of these genes inside euchromatin domain [32]. In terminally differentiated somatic cells, Hox genes are permanently silenced by their inclusion inside heterochromatin domain. Moreover, modulation of gene expression through chromatin structure is not limited only to single genes or gene clusters. For instance, in female morphogenesis an X chromosome is silenced through its condensation inside facultative heterochromatin [33-35] (the Barr body), while the active X chromosome is included in euchromatin domain. In G_0_ phase, genes of common function can colocalize inside the nuclear space in order to share the same transcription machinery [36]. Thus, these genes may be incorporated into the same transcription factory or in close neighboring transcription factories [37,38]. It appears that these active regions are positioned between chromosome territories.

In this paper we tried to identify some structural features of gene promoters located on different chromosomes in the human genome. Our hypothesis was based on the fact that promoter sequences are more exposed to the biochemical transcription machinery and therefore may reflect the chromosome boundaries much better. Previously, approaches towards promoter analysis include motif sequences and other structural parameters, such as DNA curvature, bendability, stability, nucleosome positioning or comparison of various DNA sequences [39-46]. Nevertheless, a clear association between promoter nucleotide sequences and chromosome territories was never hypothesized. The purpose of our work was to establish a possible functional significance of promoter sequences which may explain the dynamic relationship between different chromosome territories.

## Methods

In our approach we used 1200 promoter sequences (50 random promoters from each chromosome) from Transcriptional Regulatory Element Database [47,48]. We were mainly interested in the regions flanking the putative TSS, ranging from -700b to 299b. We used Visual Basic to develop a software program for promoter analysis - called PromKappa (Promoter analysis by Kappa). The source code implementation of this program is attached to our Additional file 1. We used sliding window approach to extract two types of values, namely Kappa Index of Coincidence (Kappa IC) and (C+G)%.

### Kappa index of coincidence

The Index of coincidence principle is based on letter frequency distributions and has been used for the analysis of natural-language plaintext in cryptanalysis [49]. Kappa Index of Coincidence is a form of Index of Coincidence used for matching two text strings. However, we managed to adapt Kappa IC for the analysis of a single DNA sequence. This adaptation of Kappa IC is used for calculating the level of “randomization” of a DNA sequence. Kappa IC is sensitive to various degrees of sequence organization such as simple sequence repeats (SSRs) or short tandem repeats (STRs) [50]. The formula for Kappa IC is shown below, where sequences *A* and *B* have the same length *N*. Only if an *A[i]* nucleotide from sequence *A* matches the *B[i]* correspondent from sequence *B*, then ∑ is incremented by 1. *Q* represents the number of letters in the alphabet (in our case *Q*=4).

$$\mathrm{Kapp}a_{\mathrm{IC}}=\frac{\sum_{i=1}^{N}\left[A_{i}=B_{i}\right]}{N/Q}$$

With small changes, the same method for measuring the Index of Coincidence has been applied for only one sequence, in which the sequence was actually compared with itself, as shown below in the algorithm implementation.

 function KIC(A)

  T = 0

  N = length(A) - 1

  for u = 1 to N

   B = A[u + 1] … A[N]

    for i = 1 to length(B)

     If A[i]= B[i] then C = C + 1

    next i

   T = T + (C / length(B) × 100)

   C = 0

  next u

  IC = Round((T / N), 2)

 end function

Where *N* is the length of the sliding window, *A* represents the sliding window content, *B* contains all variants of sequences generated from *A* (from *u+1* to *N*), *C* counts the number of coincidences occurring between sequence *B* and sequence *A*, and *T* variable counts the total number of coincidences found between sequences of *B* and the sequence *A*.

### Cytosine and guanine content

We extracted C+G values from each sliding window considering the nucleotide frequencies from the entire promoter sequence. In the first stage, to determine the (C+G)% content for the entire promoter sequence we used the formula:

$$CG_{\mathrm{TOT}}=\left(\frac{100}{{\left(A+T+C+G\right)}_{\mathrm{TOT}}}\right)\times {\left(C+G\right)}_{\mathrm{TOT}}$$

Where “TOT” (total) designates the promoter sequence. *CG*_*TOT*_ represents the percentage of cytosine and guanine, *(A+T+C+G)*_*TOT*_ represents the sum of occurrences of A, T, C and G, and *(C+G)*_*TOT*_ represents the sum of occurrences of C and G. In the next stage we used the value of *CG*_*TOT*_ to calculate the (C+G)% content from the sliding window (SW):

$$CG_{\mathrm{SW}}=\left(\frac{CG_{\mathrm{TOT}}}{{\left(A+T+C+G\right)}_{\mathrm{SW}}}\right)\times {\left(C+G\right)}_{\mathrm{SW}}$$

Where *CG*_*SW*_ represents the percentage of cytosine and guanine from the sliding window. In this stage, *CG*_*SW*_ value is relative to *CG*_*TOT*_. The expression *(A+T+C+G)*_*TOT*_ represents the sum of occurrences of A, T, C and G from the sliding window sequence. *(C+G)*_*SW*_ represents the sum of C and G occurrences in the sliding window sequence. Nevertheless, in our implementation we also included the option to extract *CG*_*SW*_ values without considering *CG*_*TOT*_.

### Promoter analysis

**Figure 1:**
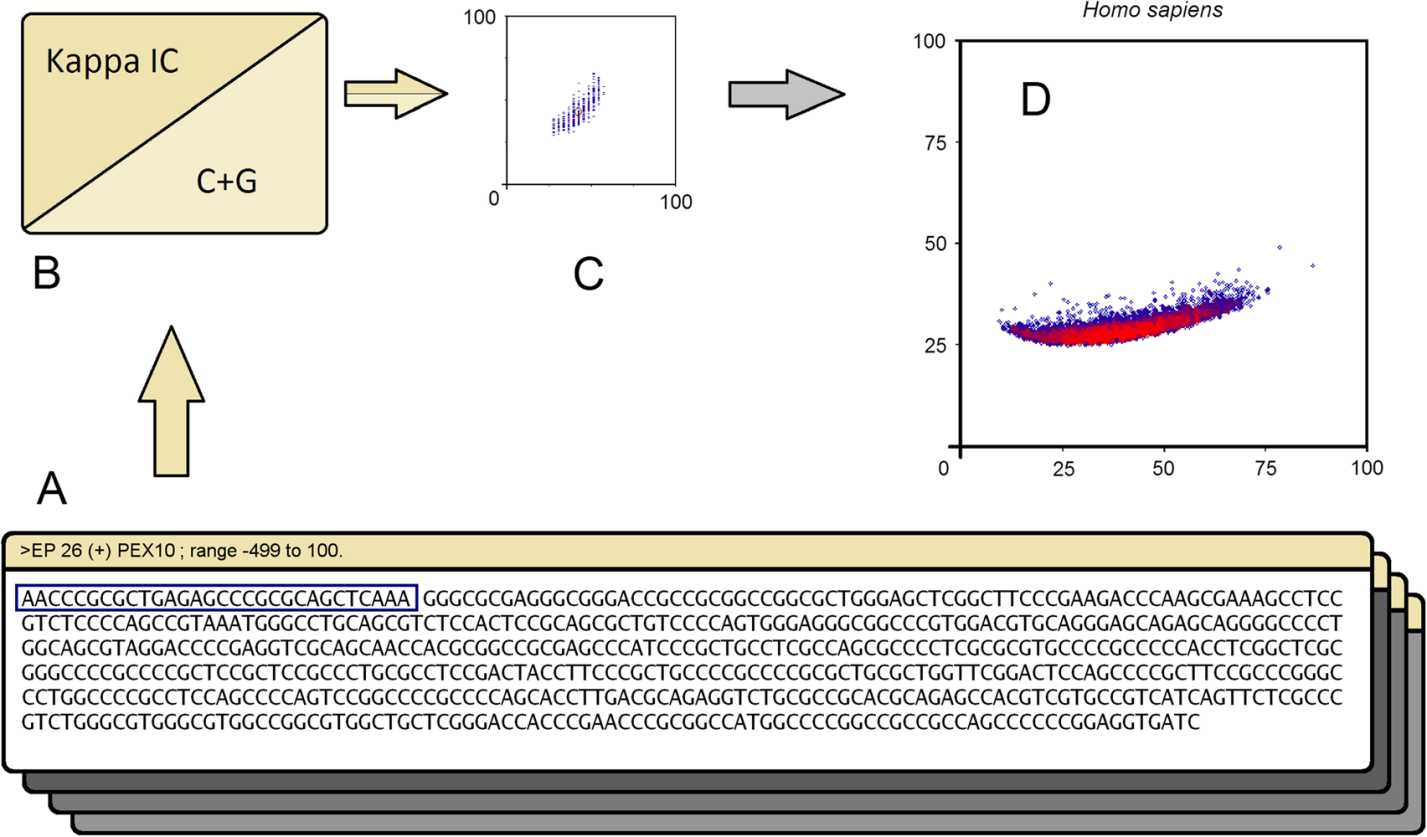
**DNA pattern analysis of promoters.** (**A**) promoter sequences, (**B**) Kappa IC and (C+G)% values extracted from each sliding window, (**C**) image-based promoter patterns generated using Kappa IC and (C+G)% values, (**D**) general distribution of promoters using the center of weight of each promoter pattern. Red color areas represent denser clusters of promoters.

By extracting Kappa IC percentages and C+G content from a sliding window (window size of 30 nt and a step of 1 nt) we have been able to measure the localized values along the promoter sequences (Figure 1A,B). Kappa Index of Coincidence values were plotted on a graph against (C+G)% values, which form a recognizable pattern for each promoter sequence (Figure 1C). The x-coordinate of each point was represented by a (C+G)% value and the y-coordinate was represented by a corresponding Kappa IC value. As expected, by using a large window size we obtained smooth promoter patterns, whereas a small window size generated sharp and distinguishable characteristics of promoters. These patterns are composed from clusters of various sizes on the y-axis (Figure 1C and Additional file 2). The center of weight from each pattern was plotted on a graph designed to show the distribution of promoters for each chromosome. Furthermore, in order to observe the boundaries in which *Homo sapiens* promoters are included, we used 8,515 gene promoters from EPD [51,52] (Eukaryotic Promoter Database) to perform a more general distribution (Figure 1D and Additional file 3). In this case we used a color scheme to highlight the denser surfaces. Red areas represent clusters of similar promoters while blue areas represent unique or rare promoters.

**Figure 2:**
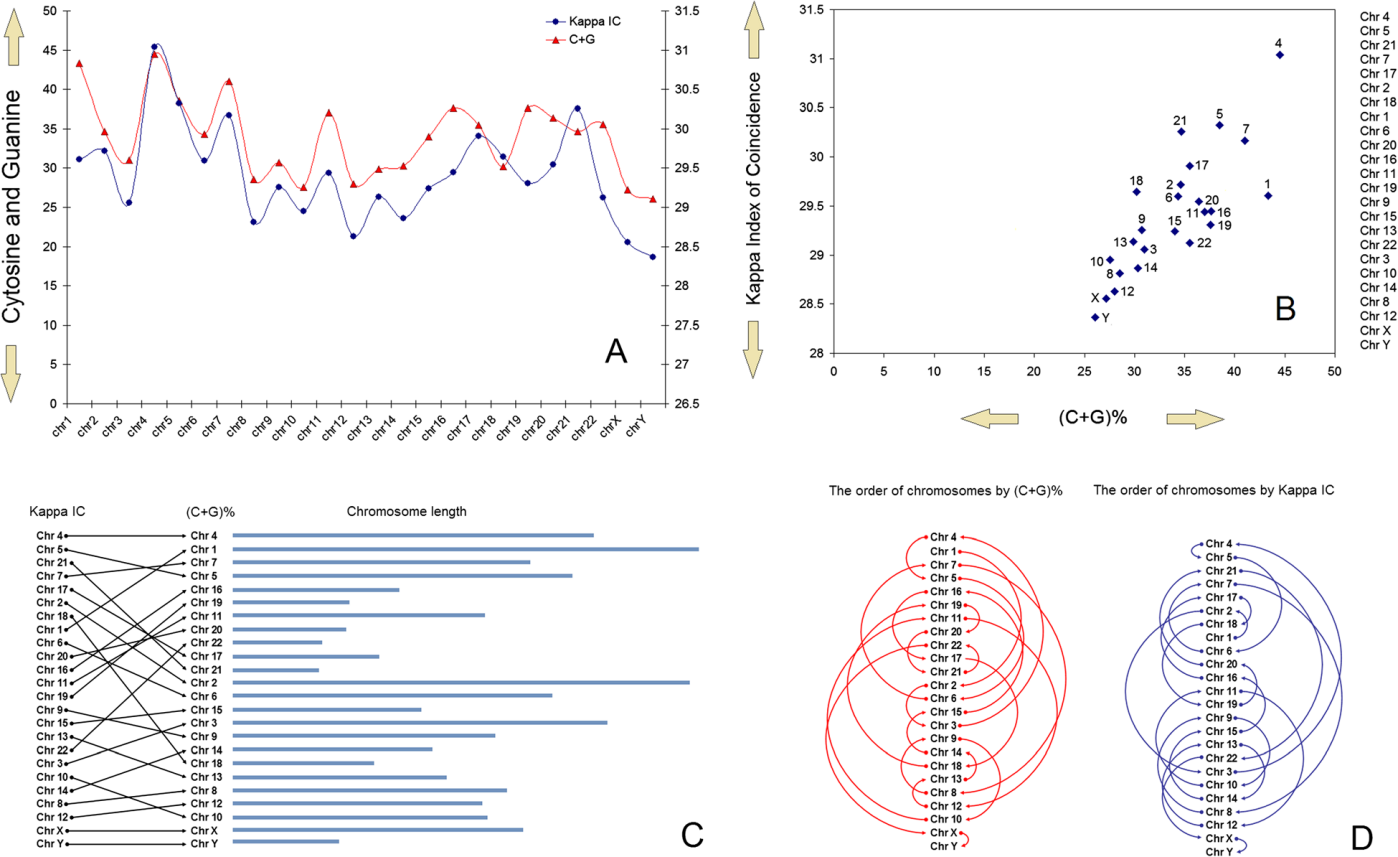
**An overall promoter-chromosome specificity and chromosome vicinities.** (**A**) red line shows the (C+G)% content for promoter sequences of each chromosome while in parallel**,** the blue line shows the value of Kappa IC for promoters of each chromosome, (**B**) diamond-shaped blue points show the position of each chromosome according to the content of (C+G)% (y-axis) and Kappa IC values (x-axis), (**C**) shows the correspondence between the order of chromosomes after Kappa IC and (C+G)% values of promoters. Light blue bars show**s** the relative length of chromosomes when they are ordered by (C+G)% values of promoters, (**D**) red arrows show the order of chromosomes by Kappa IC while blue arrows show the order of chromosomes after (C+G)% values.

## Results

We first investigated if some promoter patterns occur more often on certain chromosomes. Secondly we determined if chromosome territories could be revealed by using Kappa IC. In the third analysis we examined the distribution of Kappa IC values against the number of genetic diseases associated with each chromosome.

### Gene promoters show chromosome-specificity

Initially, our first observation regarding promoter-chromosome specificity originated from a direct correlation between their Kappa IC values and (C+G)% (Additional file 4). For the majority of chromosomes, promoter regions show almost proportional Kappa IC and CG% values relative to each other (Figure 2A). Promoters with the largest Kappa Index of Coincidence are placed on chromosome 4, while promoters from chromosomes 11 and 16 have almost the same Kappa index of coincidence and relatively close variations of cytosine and guanine content. Promoters with the lowest index of coincidence are located on chromosome Y (Figure 2B). The order of chromosomes by promoter Kappa index of coincidence is shown in Figure 2C,D. Interestingly, chromosomes X and Y contain promoters with the lowest CG% and Kappa index of coincidence values. Promoter regions with the highest Kappa Index of Coincidence values (ie. chromosomes 4,5,7,21) contain various SSRs and STRs structures (Figure 2B). This further suggests that in their evolution, promoters located on these chromosomes experienced few point mutations and accumulated more Slipped Strand Mispairing (SSM) mutations [53].

In contrast, promoter regions with the lowest Kappa Index of Coincidence values (ie. chromosomes Y,X,12,8), contain more interspersed nucleotides (A,T,C,G ≈ 25%) and less SSRs and STRs structures (Figure 2B). Acordantly, this further suggests that in their evolution, promoters located on these chromosomes have accumulated a multitude of random point mutations, thus disrupting SSR structures like poly(dA:dT) or poly(dC:dG) tracts [54,55] in shorter elements. Although without immediate consequences, point mutations that occur in promoter regions, gradually change gene expression patterns and consequently, their gene relation within certain biological pathways.

### Heterochromatin and euchromatin are two main evolutionary forces

**Figure 3:**
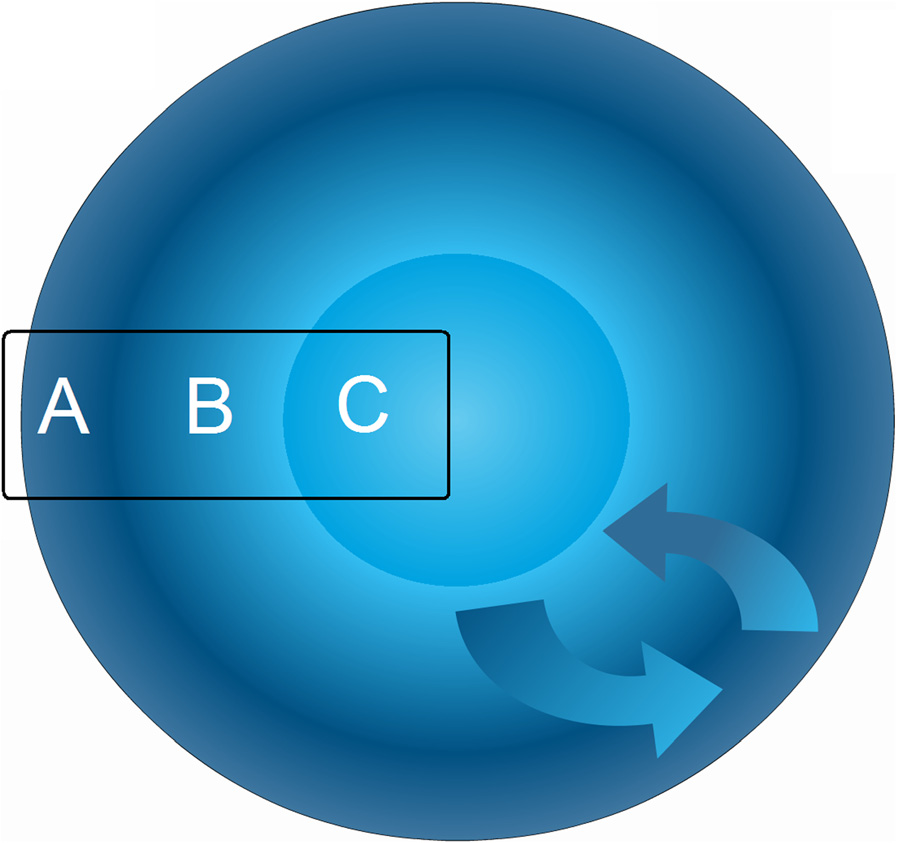
**Recycle hypothesis.** (**A**) dark blue - heterochromatin domain, (**B**) light blue - euchromatin domain, (**C**) light blue circle in the middle – the nucleolar organizing regions. Blue arrows suggest the exchange of newly formed SSRs from A, with degraded SSRs from B.

Chromosomes such as 1, 9, 16 or the Y-chromosome contain large regions of constitutive heterochromatin [56-58]. In terms of evolution, across generations the X-chromosome is also occasionally a part of heterochromatin (the Barr body). Our results suggest that promoters located on chromosomes which contain regions frequently included in heterochromatin, seem to exhibit only average to low Kappa Index of Coincidence values (Figure 2B), which further suggests that among other roles, heterochromatin is also acting as a shield for the inner core against point mutations originating from outside the nucleus. Although controversial, the “bodyguard” model [59] of heterochromatin appears to be partially true, but not as a protective role, but rather as a layered evolutionary mechanism in which some vital regions of the genome are exposed for rapid phenotypic changes (ie. tissue-specific genes) and those regions which need less change are more protected (ie. housekeeping genes). It is known that mammalian housekeeping genes evolve more slowly than tissue-specific genes [60]. Furthermore, is also accepted that non-coding regions suffer more mutations than coding regions [61]. Evolutionary, chromatin structure may influence the distribution of point mutations or other mutational events in the promoter sequence. A chromatin-dependent distribution of point mutations can lead to a gradual shift in gene expression. Gene promoters located mainly inside euchromatin domain remain prone to stable SSM mutations, favoring the maintenance of SSR or STR structures in the promoter regions. For instance, poly(dA:dT) tracts inside promoters were often associated with high gene expression levels while a disruption of poly(dA:dT) tracts in shorter elements had an opposite effect [62]. Although SSM mutations may appear with an equal probability in all promoters during DNA replication, it seems that only SSRs or STRs of promoters stored inside euchromatin are preserved. Accordingly, functional SSRs or STRs of promoters stored inside heterochromatin are gradually deteriorated by point mutations events. In most organisms, constitutive heterochromatin is usually associated with chromosomal areas of repetitive DNA sequences (commonly around the chromosome centromere and near telomeres), which seem to confer an overall trigger pattern for a tight colloid-like formation between nucleosomes [63,64]. However, functional areas (promoters and genes) that have a lower predisposition for a tight nucleosome packing, are more susceptible to point mutations inside heterochromatin than classical repetitive DNA sequences. Based on the overall promoter-chromosome specificity distributions (Figure 2), our hypothesis for a possible evolutionary dynamics of the eukaryotic nucleus would imply a permanent exchange of DNA areas between heterochromatin and euchromatin domains (Figure 3). Inside heterochromatin (Figure 3A), DNA repetitions degraded by point mutations lose their overall ability for tight nucleosome packing. Inside euchromatin (Figure 3B), SSM mutations favor DNA repetitions, which over time, gain a predisposition for tight nucleosome packing, and ultimately, allowing for heterochromatin formation. Nevertheless, in such a hypothesis the selection pressure may decide the speed by which some DNA areas are brought to the surface into the heterochromatin landscapes.

### Chromosome territories in humans

What surprised us in particular, was the symmetry of chromosome order when they are arranged by promoter Kappa IC values (Figure 2D – blue “amphora” shaped semi-circles). Generally, chromosomes were numbered according to their size. In Figure 2D we show an abstracted model in which chromosomes are ordered by Kappa IC values of promoters (colored in blue), however, in this model the blue arrows follow the order of chromosomes according to their size (starting from chromosome 4 - which contains promoters with the highest Kappa IC values). Thus, the arrows that connect more distant chromosomes in this order, show a proportional increased semi-circle radius (a radius proportional with the relative distance between them). Nevertheless, the apparent 2-fold symmetry on Y-axis (between chromosomes 4–11 and chromosomes 19-Y) further suggests that there is a correlation between chromosome length and the structure of gene promoters located on them (Figure 2D and Additional file 5). In addition, by complying with the same rules described above, when chromosomes were ordered by (C+G)% values of promoters, we could not observe any obvious symmetries (Figure 2D - red color arrows). Figure 2C shows the order of chromosomes and their position to one another when they are arranged separately by the two values.

**Figure 4:**
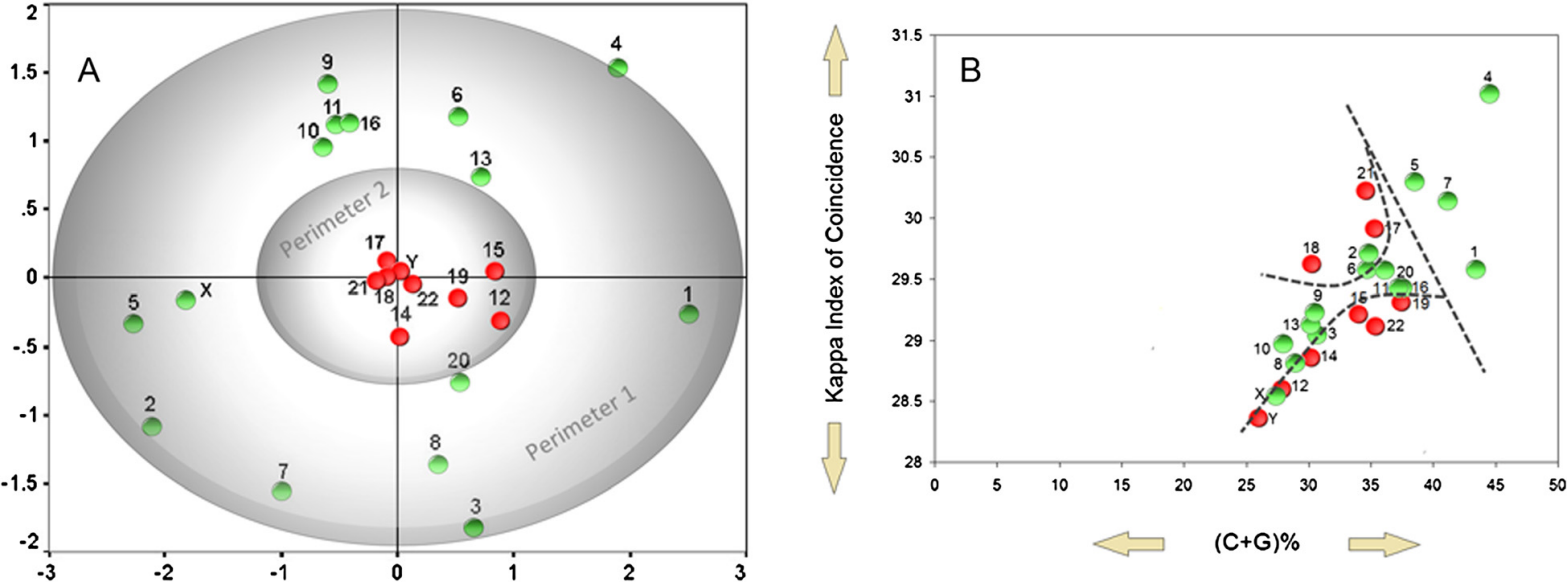
**Comparison of observed chromosome vs. general predicted positions.** (**A**) experimental results taken from human fibroblast nuclei in G0 phase by Bolzer et al., (**B**) Green and red dots show the position of each chromosome according to the content of (C+G)% (y-axis) and Kappa IC values (x-axis). The peripheral dots (red color) from panel B correspond to perimeter 2 area from panel A, whereas central dots (green color) from panel B correspond to perimeter 1 from panel A. The curved dotted lines delimit the red from the green dots to show the correlation with Bolzer et al. distribution. Diagonal dotted line shows the correlation with Lieberman-Aiden E et al. observation regarding chromosomes 16,17, 19, 20, 21 and 22.

**Figure 5:**
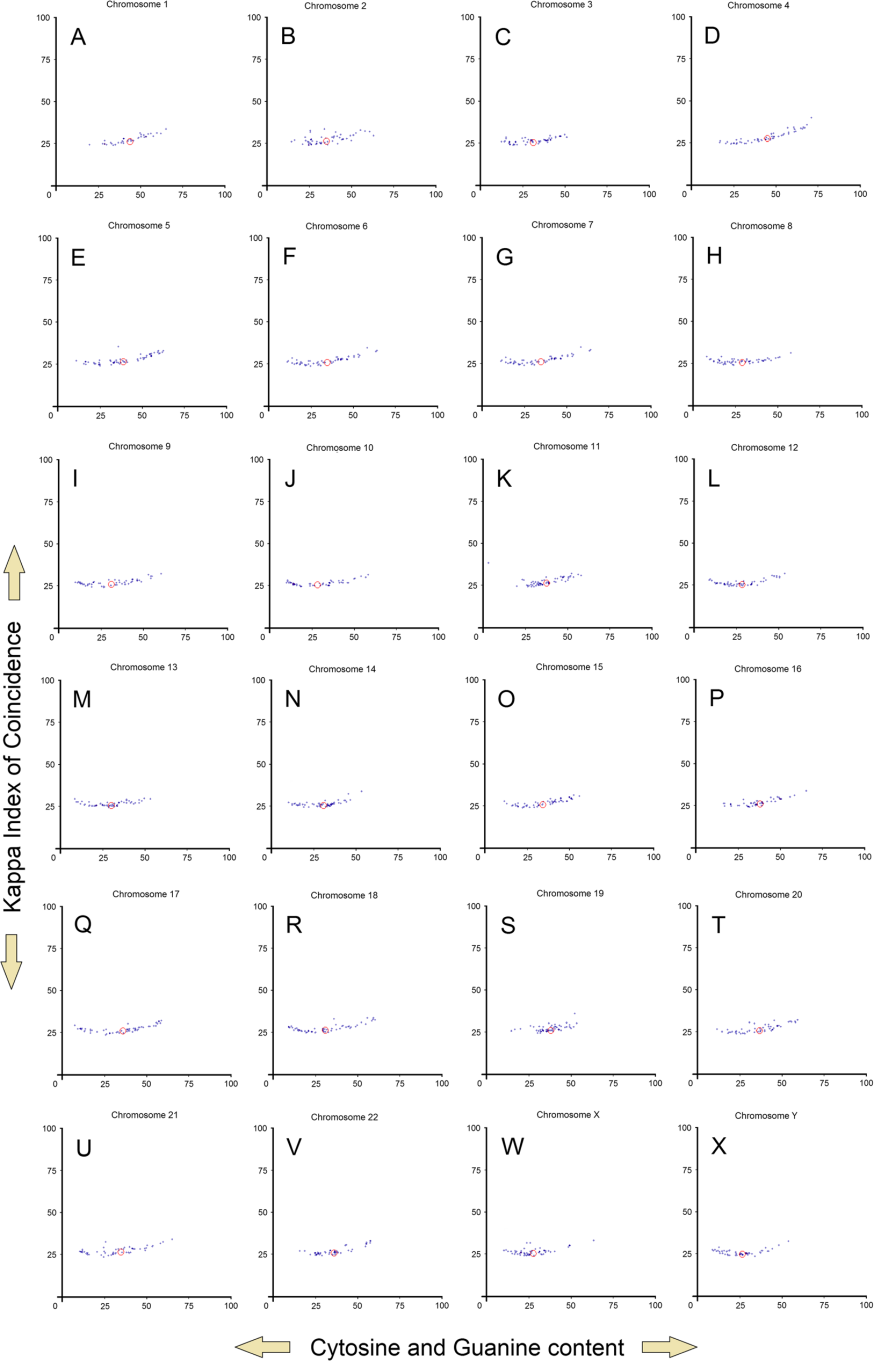
**Promoter distribution for each chromosome.** (**A-X**) Each blue point represents the center of weight from a promoter pattern belonging to chromosomes 1 up to Y. Red circles represent the blue points center of weight.

Chromosomal territories have cell-type specificity [65]. Relying exclusively on sequence composition, our promoter distributions may show which chromosomes are most frequently adjacent inside the nucleus in G_0_ phase. Human genome codes for ~2600 transcription factors [66]. However, the number of available transcription factors (and consequently the number of transcription factories) expressed at any given time is relative to each cell type. Genes located relatively close to each other in the nuclear space have a greater probability of being incorporated into the same transcription factory [67,68]. In this regard, our results suggest that gene promoters with similar structures (ie. similar DNA-binding sites and SSRs), seem to be included in the same transcription factories. This further implies that genes with different promoter structures, although close in the nuclear space, may be included in different transcription factories. Interestingly, the order of chromosomes after Kappa IC values of promoters, partially coincide with chromosomal territories of human fibroblast nuclei in G_0_ phase observed by Bolzer et al. [69] (Figure 4A). The MDS (multidimensional scaling) plot from Bolzer et al. provides a 2D distance map of the mean locations of the IGCs (fluorescence intensity gravity centers) of all heterologous chromosome territories (CTs) established from 54 G_0_ nuclei. Here, we notice some similarity of distribution for certain groups of chromosomes, such as chromosome 1 and 4 or chromosome 11 (containing beta globin gene clusters) and 16 (containing alpha globin gene clusters) (Figure 4A,B). In order to obtain an overview of this correlation with the results presented by Bolzer et al. regarding the mean locations of chromosomes in G_0_ phase (Figure 4A), we have subdivided their distribution into two main sectors. We have chosen two circular perimeters, the first perimeter (perimeter 1), which incorporates the chromosomes found at the extremity of their distribution, and a smaller circular perimeter (perimeter 2), which includes the chromosomes that are closer to the zero point (the middle of the chart). In our distribution (Figure 4B), we correlated all points present in perimeter 1 by using green dots and all points present in perimeter 2 by using red dots. We noticed that peripheral dots (red color) from our distribution correspond to perimeter 2 area from Bolzer et al. distribution, whereas central dots (green color) from our distribution correspond to perimeter 1 from Bolzer et. al distribution. Furthermore, the interchromosomal contact probabilities between pairs of chromosomes presented by Lieberman-Aiden E et al. [70], showing that chromosomes 16, 17, 19, 20, 21 and 22 preferentially interact with each other, were also correlated with our results. In our distribution of gene promoters, these chromosomes are located very close to each other and are relatively united by a single diagonal line (except chromosome 22 which is slightly below chromosome 19 – see Figure 4B), suggesting a similar conclusion. Although many factors may be involved, this comparison of observed vs. calculated positions suggests that the DNA sequence composition dictates the overall positions of chromosomes in G_0_ phase. In this regard, areas of chromosomes that contain gene promoters with common structures (ie. Kappa IC and (C+G)% values) seem to position themselves next to each other, relative to each cell type. A more detailed distribution of promoters belonging to each chromosome is shown in Figure 5, which may further detail the chromosomal areas of interaction.

### Promoter Kappa IC values vs. genetic diseases

**Figure 6:**
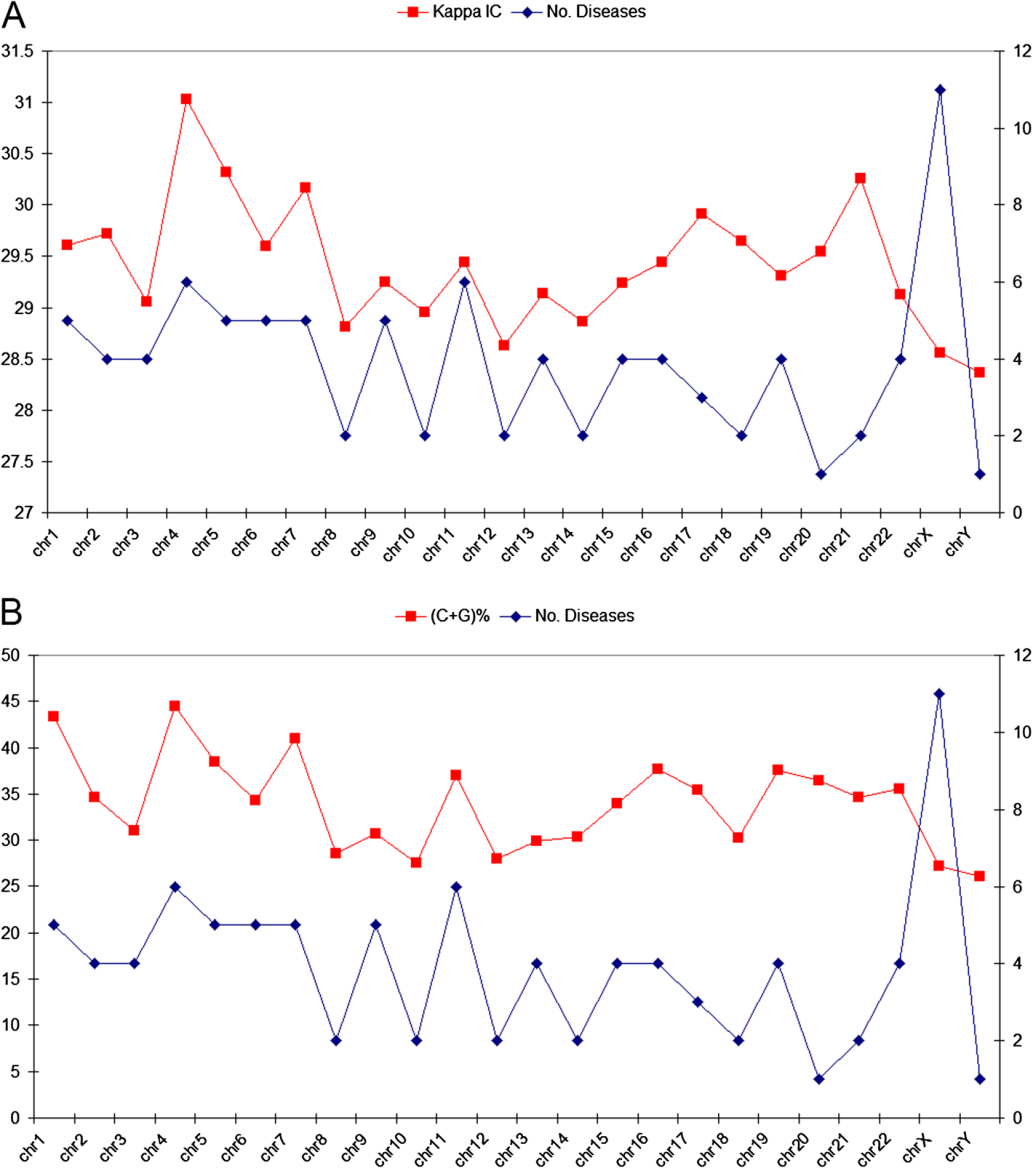
**The number of genetic diseases vs. promoter Kappa IC and (C+G) values.** (**A**) the number of genetic diseases/chromosome compared with promoter Kappa IC values/chromosome, (**B**) the number of genetic diseases/chromosome compared with promoter (C+G) values/chromosome. The scale on the right indicates the number of genetic diseases/chromosome.

A more intriguing association was made between the number of genetic diseases/chromosome and promoter Kappa IC and (C+G) values (Figure 6A,B). Although the number of genetic diseases associated with individual chromosomes may exceed several hundred, we used a list of common types of genetic diseases provided by NCBI [71]. It seems that high values of Kappa IC and (C+G)% of gene promoters are directly associated with the number of classic genetic diseases. Exception to this relative proportion are chromosomes 21, 22 and X, which exhibit asynchronous values between Kappa IC, (C+G) and the number of common genetic diseases/chromosome (Figure 6A,B).

## Discussion

**Figure 7:**
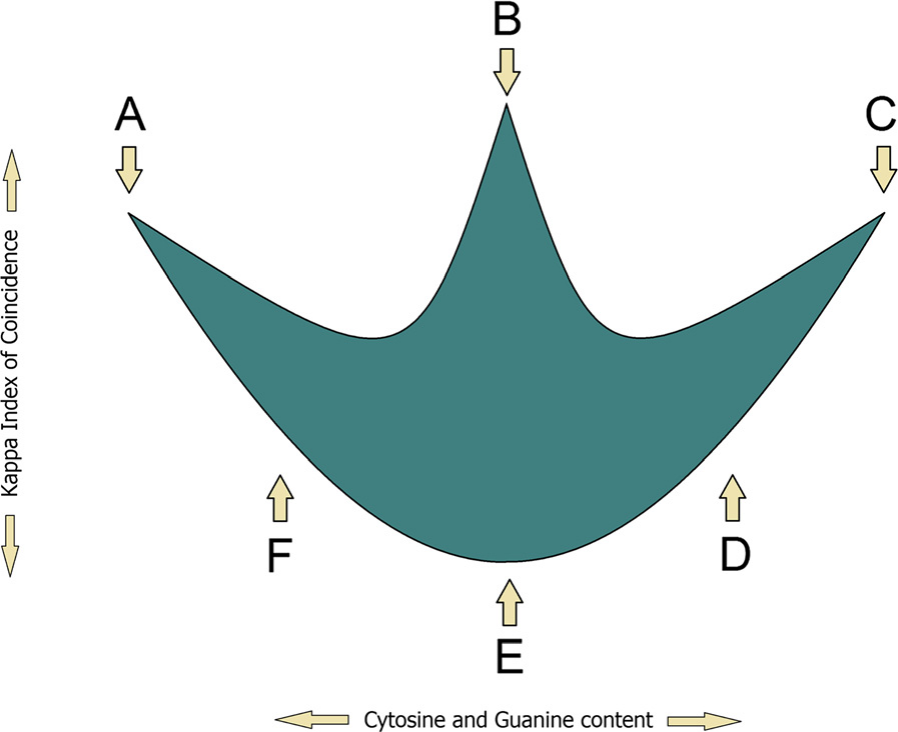
**Location of SSRs and STRs within image-based promoter patterns.** The blue shape represents a model of a promoter pattern in which we approximate the location of various structures that compose a promoter sequence. (**A**) long Poly(dA) or Poly(dT) tracts or tandem short Poly(dA) or Poly(dT) tracts, (**B**) non-ordered short Poly(dA) and Poly(dT) and Poly(dC) and Poly(dG) tracts, (**C**) long Poly(dC) or Poly(dG) tracts or tandem short Poly(dC) or Poly(dG) tracts, (**D**) short Poly(dC) and Poly(dG) tracts, (**E**) evenly interspersed nucleotides (A,T,C,G ≈ 25%), (**F**) short Poly (dA) and Poly(dT) tracts.

Gene promoters are located upstream of TSS (Transcription Start Site). A typical promoter region consists of a core promoter and regulatory domains. The association of transcription factors within a promoter precedes the RNA synthesis [72]. Accordingly, the structure of a promoter is recognized by the presence of known promoter elements, such as TATA box, GC-box, CCAAT-box, BRE and INR box [73]. In order to elucidate the evolutionary relationships, many comparisons have been made between gene promoters of different species. Nevertheless, correlations made between promoters of genes located on different chromosomes of the same species have been poorly studied. In this regard, we have chosen a different approach to analyze promoter sequences by using two-dimensional image-based patterns obtained through Kappa Index of Coincidence (Kappa IC) and (C+G)% values [74]. Each pattern is composed of vertically aligned clusters of Kappa IC (y-axis) and (G+C)% (x-axis) values. Vertical positions of these clusters form a promoter pattern which has a specific form for each promoter sequence. Their shape is explained by the presence of different structures such as simple sequence repeats (SSRs) or short tandem repeats (STRs). In order to investigate a possible relationship between promoters of genes located on different chromosomes, we have plotted the center of weight from 1200 promoter patterns (Figure 5A-X). The center of weight of each promoter pattern indicates an average between all SSRs and STRs present in the promoter sequence. An explanatory model of an image-based promoter pattern can reveal some visual insights into different promoter regions, such as the locations of all SSRs and STRs (Figure 7A-F). We have also noticed the directions and the angles of these promoter distributions which may suggest an evolutionary tendency (Figure 1D).

The haploid human genome contains a nuclear volume of approximately 1000 μm^3^ and 3.2 billion base pairs of compacted DNA [75-77]. Nucleosomes compact and regulate access to DNA by assuming specific positions [78,79]. The interaction between nucleosomes that incorporate functional sequences located at great distances inside the nucleous, is provided by a favorable positioning of other nucleosomes that incorporate non-coding sequences. Accordingly, an overall picture begins to take shape, namely that the evolutionary process can not tolerate non-functional information. Although many studies show that refined mechanisms involved in the dynamics of the nucleus are ATP (adenosine-5'-triphosphate) dependent processes [80,81], we wonderd if self-organization processes and other biophysical phenomena could be evan more involved than previously thought. Nevertheless, DNA guided self-organization processes that may concern chromatin mobility will be of utmost importance for our understanding of the dynamics of the nucleus.

In a recent study, we have suggested that eukaryotic genomes may exhibit at least 10 classes of promoters [82]. In future research we wish to highlight the distribution of these promoter classes on each chromosome. Furthermore, we are also interested to observe the differences between Kappa IC values of introns and exons related to each chromosome in order to understand if the relative proportions presented here will remain constant.

## Conclusions

In this paper a comprehensive analysis was undertaken for promoter sequences from *Homo sapiens*. In our approach we used 1200 promoter sequences (50 random promoters from each chromosome) from Transcriptional Regulatory Element Database. In order to measure the structural similarity of gene promoters, we used two-dimensional image-based patterns obtained through Kappa Index of Coincidence (Kappa IC) and (C+G)% values. The center of weight of each promoter pattern indicated an average between all SSRs and STRs present in the promoter sequence. A distribution of these average values showed that gene promoters appear to be specific to each chromosome. Furthermore, the proximity between chromosomes seems to be in accordance to the structural similarity of their gene promoters. Although chromosomes are positioned differently depending upon each cell type, they exhibit a predisposition for a standard arrangement. High Kappa IC and (C+G)% values of gene promoters were also directly associated with the most frequent genetic diseases. Taking into consideration these observations, a general hypothesis for the evolutionary dynamics of the genome has been proposed. In this hypothesis, heterochromatin and euchromatin domains exchange DNA sequences according to a difference in the rate of mutations.

## Competing interests

The authors declare that they have no competing interests.

## Authors’ contributions

PG conceived of the study and participated in its design and coordination. PG created the algorithms and the software used in the analysis. CIT carried out the assembly of chromosome specific promoter files and manually tested the correctness of each promoter sequence. PA and CIT participated in the promoter sequence analysis and drafted the manuscript. Both authors have verified the accuracy of the data. Both authors read and approved the final manuscript.

## Supplementary Material

Additional file 1 Binaries and source code files of PromKappa (Promoter analysis by Kappa Index of Coincidence) software used for promoter pattern analysis. Click here for file

Additional file 2 Examples of image-based promoter patterns. Click here for file

Additional file 3 A complete set of 8,512 gene promoters from The Eukaryotic Promoter Database. Click here for file

Additional file 4 Distribution of numerical values. Click here for file

Additional file 5 Chromosomes ordered by Kappa IC and (C+G)% mean values of their gene promoters. Click here for file
