# Supplementary material for: Gene promoters show chromosome-specificity and reveal chromosome territories in humans
Source: BMC Genomics. 2013 Apr 24;14:278. doi: 10.1186/1471-2164-14-278 (PMC3668249; doi:10.1186/1471-2164-14-278)
Supplement: Additional file 2 — Examples of image-based promoter patterns. [file 1471-2164-14-278-S2.doc]

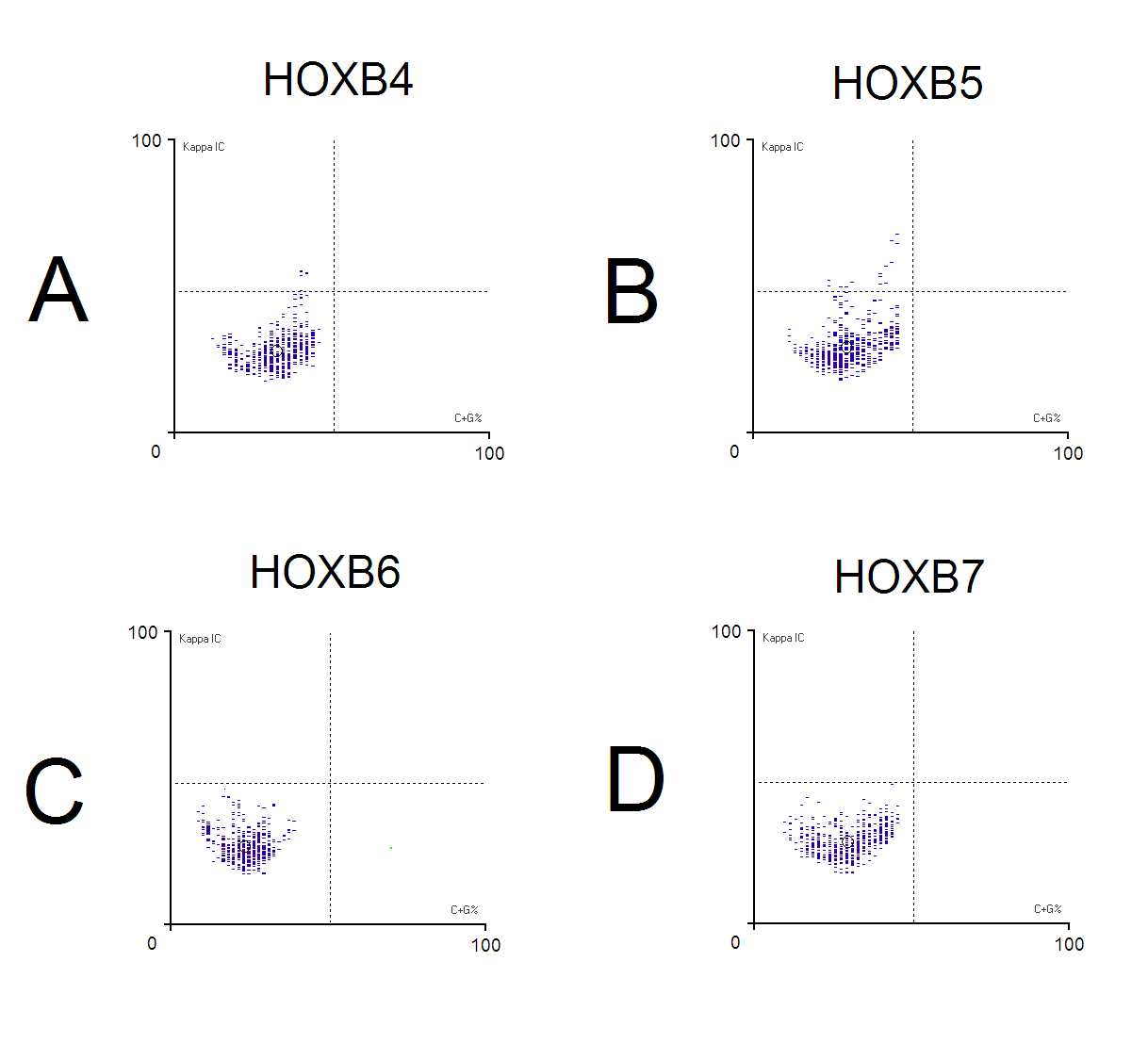


Figure 8. Examples of image-based promoter patterns from HOXB gene promoters.


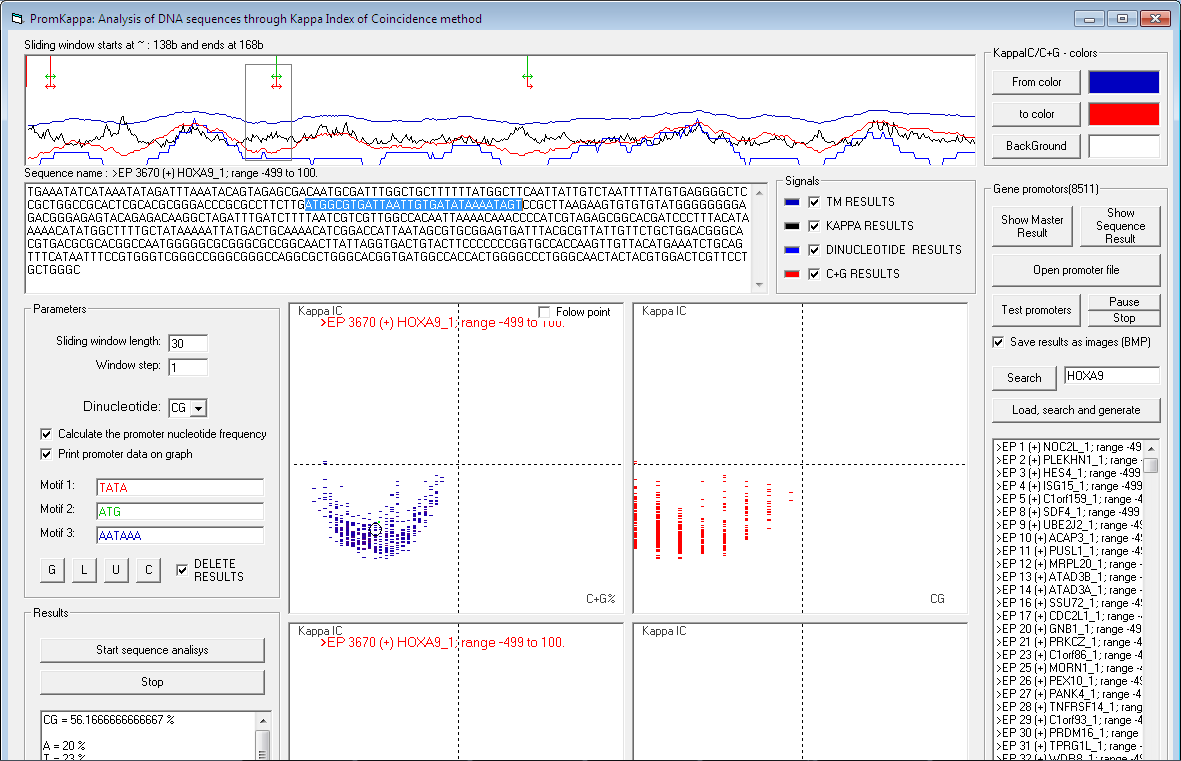


Figure 9. PromKappa ScreenShot - generating an image-based pattern of HOXA3 gene promoter.
