# Supplementary material for: Gene promoters show chromosome-specificity and reveal chromosome territories in humans
Source: BMC Genomics. 2013 Apr 24;14:278. doi: 10.1186/1471-2164-14-278 (PMC3668249; doi:10.1186/1471-2164-14-278)
Supplement: Additional file 5 — Chromosomes ordered by Kappa IC and (C+G)% mean values of their gene promoters. [file 1471-2164-14-278-S5.ppt]

## Slide 1
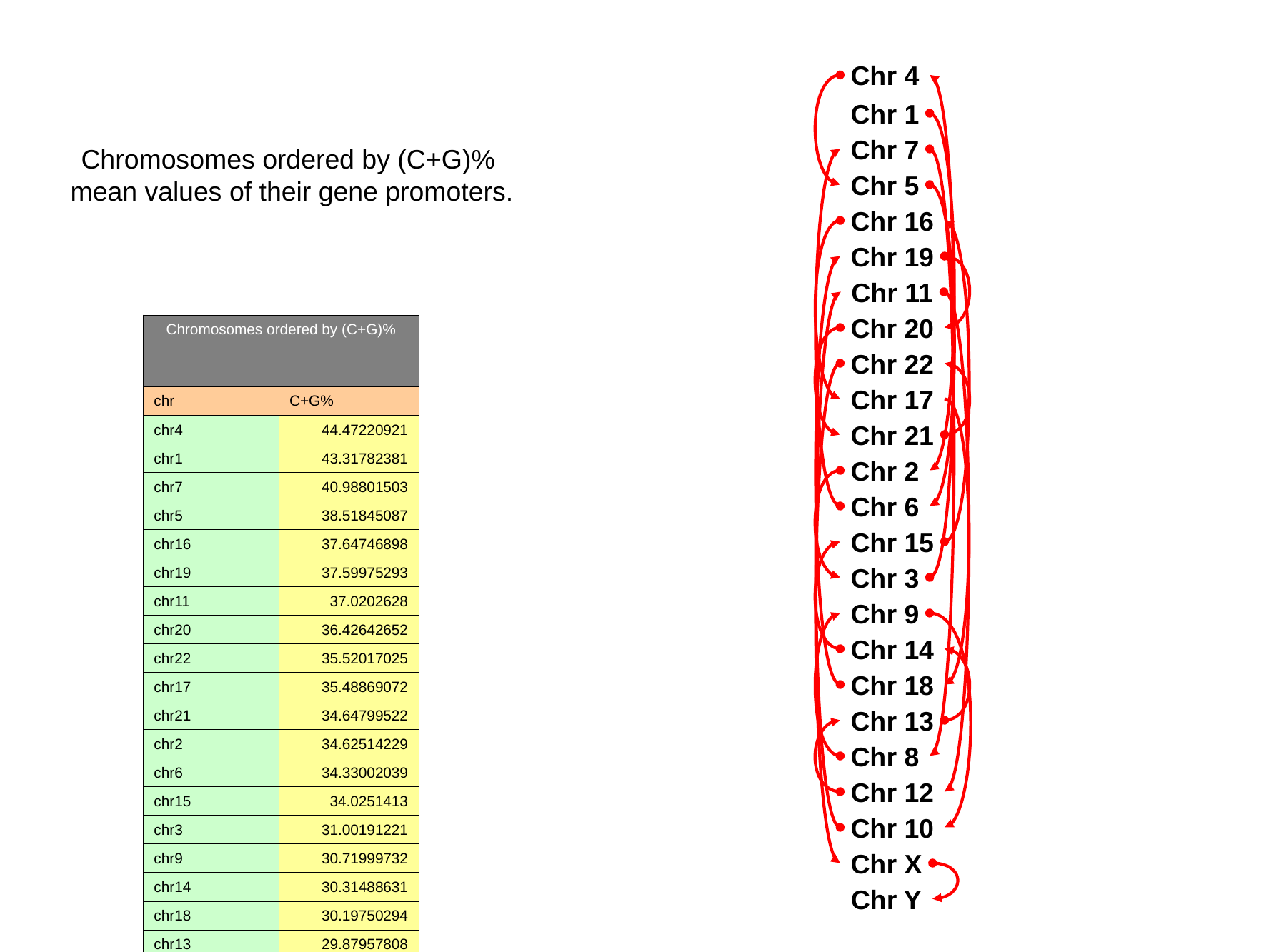

Chr 4
Chr 1
Chr 7
Chromosomes ordered by (C+G)%
mean values of their gene promoters.
Chr 5
Chr 16
Chr 19
Chr 11
Chr 20
| Chromosomes ordered by (C+G)% | |
| --- | --- |
| | |
| chr | C+G% |
| chr4 | 44.47220921 |
| chr1 | 43.31782381 |
| chr7 | 40.98801503 |
| chr5 | 38.51845087 |
| chr16 | 37.64746898 |
| chr19 | 37.59975293 |
| chr11 | 37.0202628 |
| chr20 | 36.42642652 |
| chr22 | 35.52017025 |
| chr17 | 35.48869072 |
| chr21 | 34.64799522 |
| chr2 | 34.62514229 |
| chr6 | 34.33002039 |
| chr15 | 34.0251413 |
| chr3 | 31.00191221 |
| chr9 | 30.71999732 |
| chr14 | 30.31488631 |
| chr18 | 30.19750294 |
| chr13 | 29.87957808 |
| chr8 | 28.52520892 |
| chr12 | 27.99039251 |
| chr10 | 27.53173487 |
| chrX | 27.19556264 |
| chrY | 26.07920071 |
Chr 22
Chr 17
Chr 21
Chr 2
Chr 6
Chr 15
Chr 3
Chr 9
Chr 14
Chr 18
Chr 13
Chr 8
Chr 12
Chr 10
Chr X
Chr Y

## Slide 2
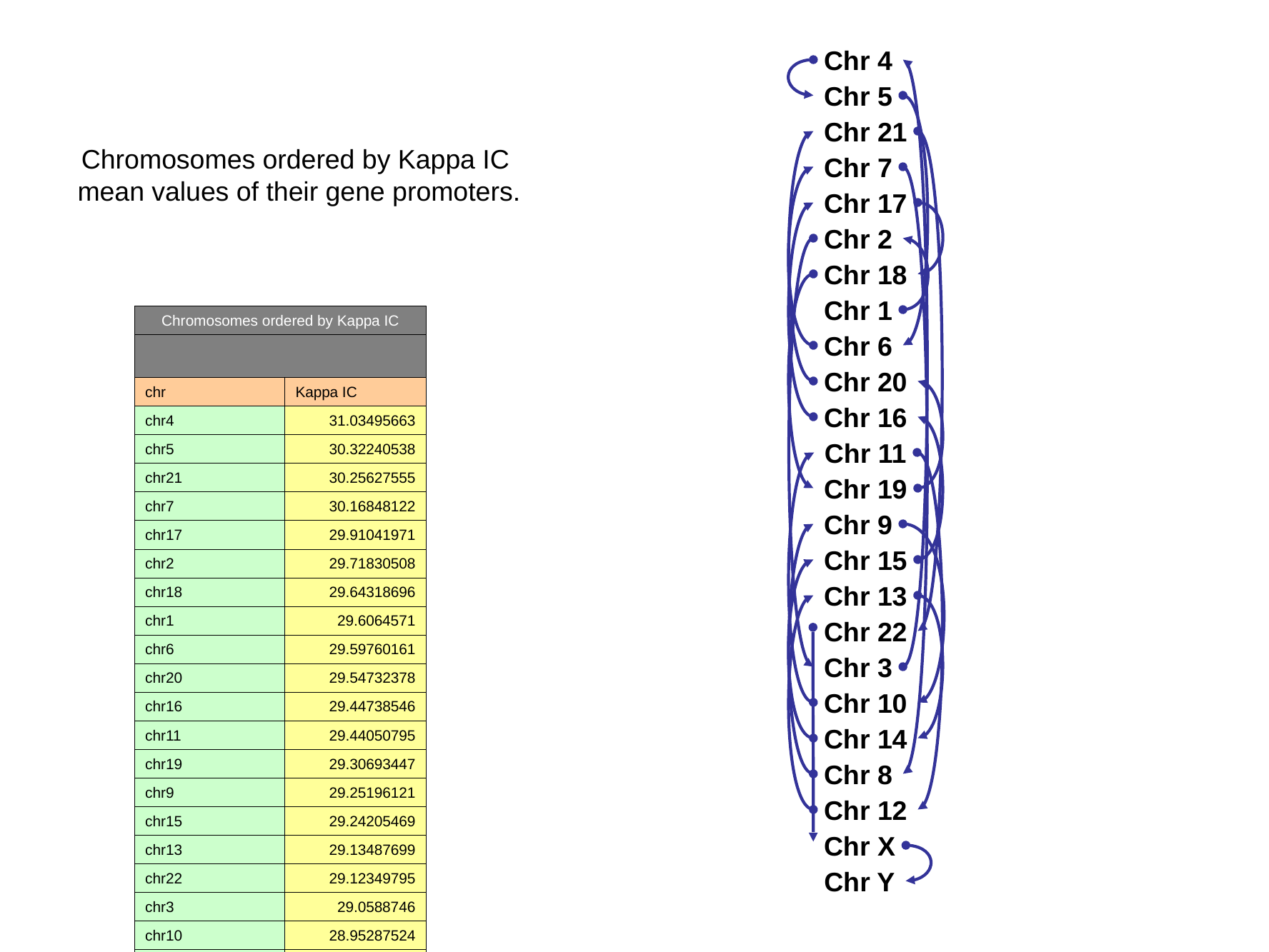

Chr 4
Chr 5
Chr 21
Chromosomes ordered by Kappa IC
mean values of their gene promoters.
Chr 7
Chr 17
Chr 2
Chr 18
Chr 1
| Chromosomes ordered by Kappa IC | |
| --- | --- |
| | |
| chr | Kappa IC |
| chr4 | 31.03495663 |
| chr5 | 30.32240538 |
| chr21 | 30.25627555 |
| chr7 | 30.16848122 |
| chr17 | 29.91041971 |
| chr2 | 29.71830508 |
| chr18 | 29.64318696 |
| chr1 | 29.6064571 |
| chr6 | 29.59760161 |
| chr20 | 29.54732378 |
| chr16 | 29.44738546 |
| chr11 | 29.44050795 |
| chr19 | 29.30693447 |
| chr9 | 29.25196121 |
| chr15 | 29.24205469 |
| chr13 | 29.13487699 |
| chr22 | 29.12349795 |
| chr3 | 29.0588746 |
| chr10 | 28.95287524 |
| chr14 | 28.86242687 |
| chr8 | 28.81054971 |
| chr12 | 28.62859672 |
| chrX | 28.55760388 |
| chrY | 28.36175328 |
Chr 6
Chr 20
Chr 16
Chr 11
Chr 19
Chr 9
Chr 15
Chr 13
Chr 22
Chr 3
Chr 10
Chr 14
Chr 8
Chr 12
Chr X
Chr Y

## Slide 3
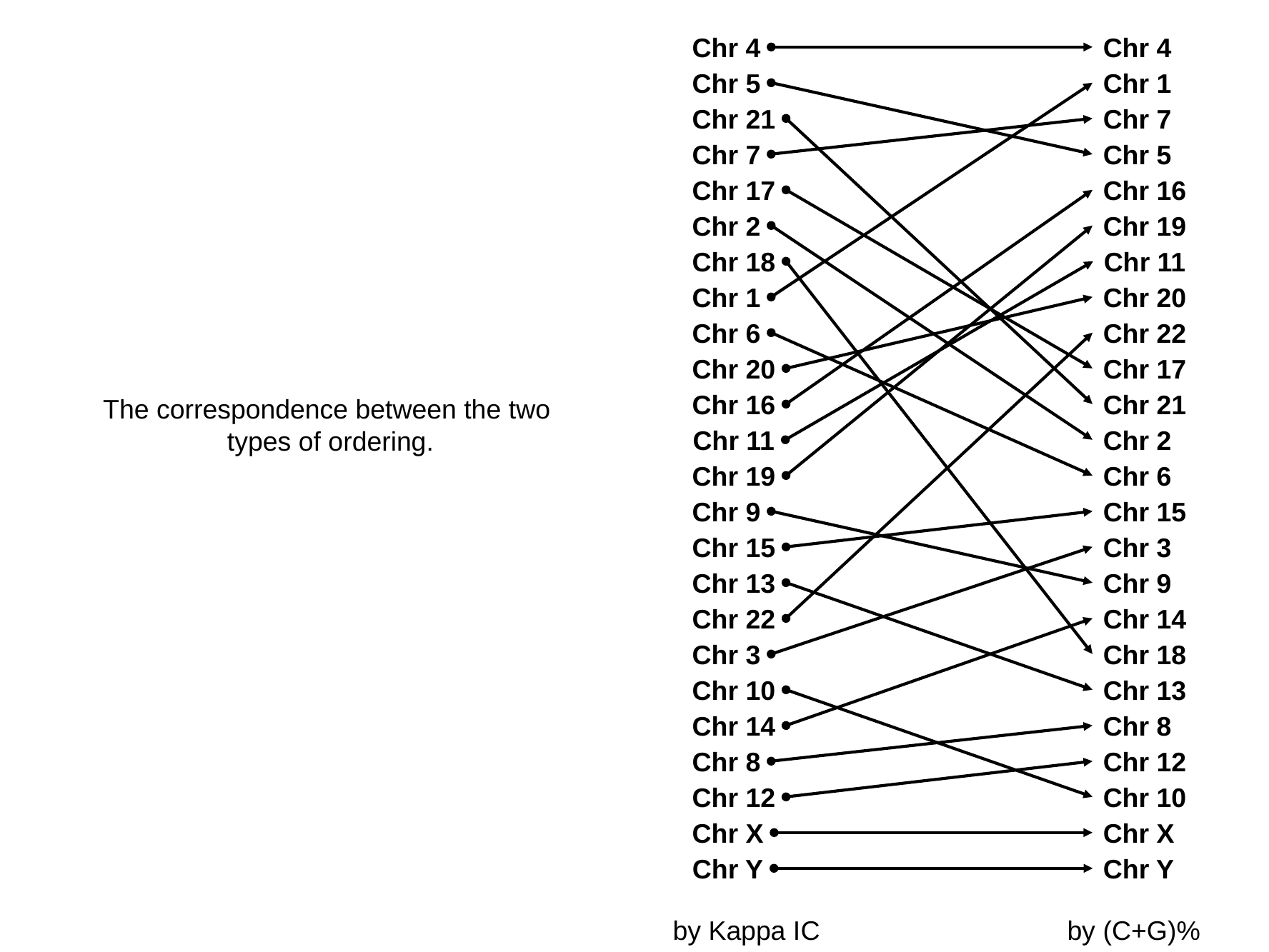

Chr 4
Chr 4
Chr 5
Chr 1
Chr 21
Chr 7
Chr 7
Chr 5
Chr 17
Chr 16
Chr 2
Chr 19
Chr 18
Chr 11
Chr 1
Chr 20
Chr 6
Chr 22
Chr 20
Chr 17
Chr 16
Chr 21
The correspondence between the two
 types of ordering.
Chr 11
Chr 2
Chr 19
Chr 6
Chr 9
Chr 15
Chr 15
Chr 3
Chr 13
Chr 9
Chr 22
Chr 14
Chr 3
Chr 18
Chr 10
Chr 13
Chr 14
Chr 8
Chr 8
Chr 12
Chr 12
Chr 10
Chr X
Chr X
Chr Y
Chr Y
by Kappa IC
by (C+G)%
