# Supplementary material for: Gene promoters show chromosome-specificity and reveal chromosome territories in humans
Source: BMC Genomics. 2013 Apr 24;14:278. doi: 10.1186/1471-2164-14-278 (PMC3668249; doi:10.1186/1471-2164-14-278)
Supplement: Additional file 1 — Binaries and source code files of PromKappa (Promoter analysis by Kappa Index of Coincidence) software used for promoter pattern analysis. [file 1471-2164-14-278-S1.zip › PromKappa ScreenShots.doc]

**Figure 1** PromKappa. (**a**) background and pattern color, (**b**) promoter pattern Tab, (**c**) promoter pattern distribution Tab (**d**) file loading, (**e**) gene name search, (**f**) automatic searching of gene names in the promoter list, (**g**) promoter sequence container, (**i**) manual analysis of a single promoter sequence, (**h**) buttons for promoter sequence editing (G-random sequence generation, L-lowercase U-uppercase, C-sequence filtering of carriage return (CR) and line feed (LF) characters), (**j**) text box containing the promoter sequence for immediate analysis.

**Figure 2** Relationship between the shape of the promoter pattern and different structures such as Simple Sequence Repeats (SSRs) or short tandem repeats (STRs). (**a**) the sliding window, (**b**) green line in the middle of the pattern reflects the sliding window position and the local characteristics of the promoter sequence.

**Figure 3** Distribution of promoters in *Mus Musculus*. (**a**) each circle represents the center of weight from a promoter pattern, (**b**) on click, the button is showing the promoter pattern distribution Tab.
